# Micro- and Nano-Plastics in Drinking Water: Threat or Hype? Critical State-of-the-Art Analysis of Risks and Approaches

**DOI:** 10.3390/jox15030085

**Published:** 2025-06-03

**Authors:** Andrea G. Capodaglio

**Affiliations:** DICAR, University of Pavia, Via Ferrata 3, 27100 Pavia, Italy; andrea.capodaglio@unipv.it

**Keywords:** freshwater, microplastics, drinking water, treatment processes, nanoplastics, health risks

## Abstract

Microplastic (MP) contamination affects all environmental media, even in remote, unpopulated regions of the globe. Many studies have addressed this issue under various aspects; however, actual and definitive evidence that MPs are a cause of human health risk in actual environmental conditions has not been provided. MP decomposition generates smaller nanoplastics (NPs) with different properties, closer to engineered nanoparticles than to MP. Their detection is more complex and laborious than MP’s, and, as such, their fate and effects are still poorly studied. Advanced technologies to remove MP/NPs from supply water are being investigated, but current evidence indicates that conventional drinking water treatment facilities efficiently remove a major part of MPs, at least as far as sizes greater than 20 µm. Notwithstanding recent developments in MP/NP classification and detection techniques, at the moment, very few studies specifically address NPs, which, therefore, deserve more targeted investigation. This paper addresses MPs and NPs in drinking water, examining recent current literature on their presence and state-of-the-art in risk assessment and toxicology. The paper also critically overviews treatment technologies for their removal and discusses the present knowledge gap and possible approaches to this widespread issue.

## 1. Introduction

The widespread use of plastic compounds as an indispensable industrial commodity began in the 1950s: their popularity was due to the fact that they are cheap to manufacture, easily molded, and light compared to alternative materials for intended uses. Studies showed that, if properly managed, plastic is highly ecologically friendly compared to possible existing alternatives: their production requires at least 2–4% less energy than current alternative materials and generates about three times less greenhouse gas (GHG). Furthermore, substituting plastic with other materials in all sectors would require, on average, about 57% more energy consumption and increase GHG emissions by 61% overall [1,2]. A Life Cycle Assessment (LCA) impact study on plastic products versus other materials conducted by global management consultants McKinsey & Company found that plastic has the lowest energy and carbon footprint in thirteen out of fourteen different examined applications. In addition to production energy emissions (Scope 2), plastic’s light weight contributes to the reduction of transport-related consumption, positively impacting indirect emissions (Scope 3). Overall, a plastic bottle provides a 15% GHG emission advantage versus a glass one and up to 50% versus an aluminum can [3]. Plastic also contributes to a circular economy by being highly recyclable: it is estimated that almost 90% of households in developed countries have access to the possibility to recycle these materials [4,5]. PET and HDPE bottles can be made from 100% recycled content; additionally, pyrolysis can convert polymeric waste into renewable, high-quality oils and chemicals without releasing toxic substances into the atmosphere [6].

**Figure 1:**
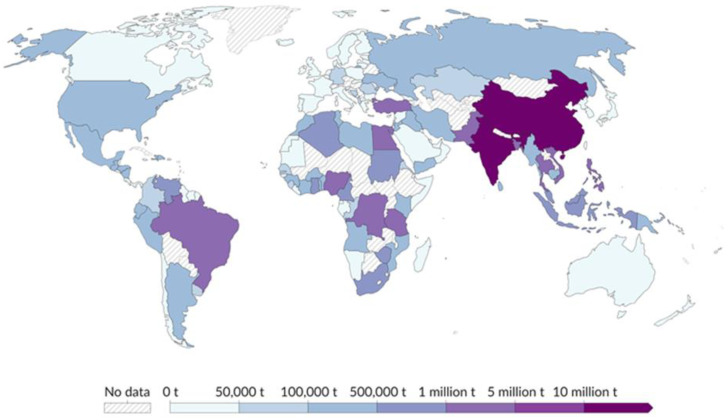
Mismanaged plastic waste, according to [16].

Since its commercial introduction, over 9 billion tons of plastic products have been produced, of which an estimated 70% were discarded, resulting in about 7 billion tons of cumulated plastic waste [7]. Often, however, plastic waste is poorly managed, i.e., dumped in unregulated landfills, in surface waters, or directly into seas, especially in developing countries (Figure 1). Its fate is largely unknown; however, estimates indicate that 4.8–12.7 Mt/y of macroplastic waste (bottles, bags, food containers, fishing gear, nets, etc.) is currently dispersed into oceans worldwide [8]. This figure, which is probably underestimated, constantly adds to the estimated 195 Mt already present in global waters [9]. Plastic is eventually subject to physical breakdown through natural processes (bio-, photo-, thermo-oxidative degradation, and hydrolytic reactions), starting at their accessible polymeric surface and accelerating progressively due to the gradually increasing specific exposed area [10]. This is usually denoted with the terms aging or weathering, indicating the change of polymer properties (composition, particle integrity, surface properties) over time [11].

An outcome of plastic waste mismanagement [12], microplastic (MP) (i.e., particles ≤5 mm, according to the mainstream definition) contamination is a ubiquitous phenomenon globally affecting even remote, pristine, high altitude and uninhabited polar regions [13,14]. MPs vary in size, characteristics, and polymeric nature, and the seven most common categories are acrylic or polymethyl methacrylate (PMMA), polycarbonate (PC), polyethylene (PE), polypropylene (PP), polyethylene terephthalate (PETE or PET), polyvinyl chloride (PVC), acrylonitrile-butadiene-styrene (ABS). Overall, there are around 200 different types of polymers, with diverse subcategories that obtain their specific properties from other chemicals (additives) to allow them to be processed into millions of products [15].

From an environmental perspective, MPs are differentiated between primary and secondary: the former already enter the environment in size ≤5 mm; they are usually specifically manufactured for industrial production purposes (drug vectors, cosmetic ingredients, industrial building blocks for final products), but their environmental immission is relatively low, estimated at 0.8–2.5 Mt/y. The largest MP fraction, i.e., secondary MPs, derives from macroplastic weathering or originates from common domestic and industrial activities, vehicle tire wear, marine coatings, road markings, city dust, and others; for example, synthetic fibers’ garment washing could contribute up to 2000 MP/garment-wash. Wastewater discharges are also point sources of MPs release into surface waters, although wastewater treatment plants (WWTPs) efficiently remove most of the original influent load: conventional units such as dissolved air flotation and sedimentation can remove up to 95% MPs (75% on average). Biological units remove MPs mainly by incorporation in biological flocs, with an average efficiency of 92%. The most effective removal is observed in membrane bioreactors (MBRs), achieving removal efficiency close to 99% [17]. It should be noted that although MPs are removed in WWTPs, they are generally not destroyed, i.e., their fate is disconnected from plant effluents but persists in other media (e.g., biological or chemical sludge).

**Figure 2:**
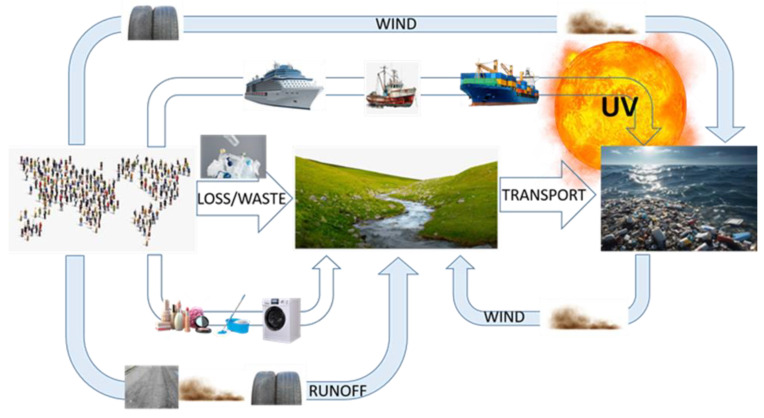
Macro and micro/nano plastics environmental cycle.

MPs have been detected in sediment, biota, agricultural soils, and air [18]; MP contamination could be compared to other instances of global transboundary pollution originating from entirely anthropogenic substances (e.g., Polychlorinated biphenyls, PCBs, Per- and poly-fluoroalkyl substances, PFASs, PBDEs, etc.) [19,20]. Weathering of macroplastic waste into MPs is faster on land than in water due to exposure to higher temperatures and sunlight irradiation; the most significant drivers of MPs transport from land into surface waters are rainfall intensity and soil erosion [21]. MPs persist in aquatic environments for long periods, susceptible to further fragmentation and dispersion: ocean waves vaporize water, salt, and pollutants, including MPs, promoting their aerosolization. Wind dispersion effectively makes oceans a relevant secondary source of atmospheric MPs [22]. Figure 2 depicts the simplified environmental cycle of macro and microplastic transport.

In time, floating plastic in marine environments (e.g., the ‘great Pacific garbage patch’, GPGP, in the central North Pacific Ocean, covering 1.6 million km^2^) have developed into ecosystems of their own, known as ‘plastisphere’, which act as colonization support for diverse aquatic and bird species, as well as vast microbial populations [23,24]. Although highly persistent, MPs could be destroyed by the latter: *Parengyodontium album* isolated from the GPGP has shown the capability of mineralizing UV-exposed PE into CO_2_ [25]. Similarly, *Bacillus cereus* can mineralize low-density polyethylene (LDPE) and PS [26]; several other bacterial and fungal strains can contribute to MP destruction through joint metabolism [27].

The aim of this paper is to critically address the issue of MP/NPs in drinking water in the light of recent literature and highlight the need for better precision in tackling this subject, to discuss the need and relevance of specific removal technologies from drinking water, and the impact of possible approaches and future research needs.

## 2. Methodology

A literature review of scientific articles, technical reports, official documents, and established standards was performed, initially limited to publications dating from 2018. The keywords “microplastics”, “nanoplastics”, “water”, “drinking water”, “detection”, “characterization”, “health effect”, and “health risk” in various combinations were used for multiple online searches on Google Scholar. Scientific articles in non-peer-reviewed journals were excluded, and only the most recent review papers were considered. Documents from sectoral industrial websites or information media were included when deemed credible and appropriate.

## 3. MPs in Aquatic Environments

MP presence in global waters has been the object of innumerable studies: a present Google Scholar search on the subject returned over 26,000 hits just since 2020. The majority of field studies on MPs in aquatic environments report the highest typical counts of ˂1 to a few 100’s MPs/m^3^: in the open sea, their mean concentration was reported as 0.031–0.305 MP/m^3^. Notwithstanding the common perception of the GPGP as consisting of a giant island of floating waste, fostered by several media showing unrelated images, its actual average MP density is just 4 MP/m^3^, which prevents its detection by satellites and even by boats crossing the area [28]. Much higher MP counts are observed in certain coastal areas: up to 6600 MP/m^3^ in the East China Sea, near the Yangtze estuary [29]. The average MP concentration in commercial ports’ inner waters ranges from 0.1 to 3 × 10^6^ MP/m^3^, with the highest numbers observed recently at the ports of Mongla (Bangladesh) at 3 × 10^6^ MP/m^3^ and Tua (Indonesia) at ≈625.000 MP/m^3^ [30]. The highest MP counts in freshwaters (>5.4 × 10^6^ MP/m^3^) have been consistently monitored in China (the world’s biggest plastic producer) and occasionally in U.S. locations. European freshwaters usually show MP concentrations between 1–100 MP/m^3^ [31].

Between different raw water sources, groundwater has shown by far the lowest microplastic concentrations [32]; however, despite the general scarcity of studies on the subject, high concentrations were observed in Chinese wells (up to 6832 MP/m^3^, with of average 2100 MP/m^3^), and in Southwest Iran (up to 1300 MP/m^3^, mean 480 MP/m^3^) [33]. Groundwater distributed as drinking supply in Germany contained up to 7 MP/m^3^, with 60% of samples showing no MPs [34]. The reason for the reported figures huge variability is not clear, but it could be assumed to be due in part to the limited number of replications in each, the different adopted methodologies among studies, or both.

MP exposure from drinking water is commonly touted by mainstream media as a potentially serious risk to human health through biotic accumulation and pollutants vector effect since 81% of tap water sampled around the globe has shown some level of contamination; however, actual counts are generally low due to the effect of conventional drinking water treatment technology: in EU countries an average of 3.6 MP/L were reported, in the US 6.2 MP/L, and in India 9.2 MP/L [35]. In comparison to bottled water, containing up to 240,000 MP/L (90% of which ≤20 µm) [36], tap water can be considered a minor contributor to human MP ingestion, estimated at 3000–6000 MP/person-y. While this may seem a large number, it ought to be compared to >20-fold assimilation from bottled water consumption (64,000–127,000 MP/person-y) and an approximate 10-fold inhalation from ambient air (35,000–62,000 MP/person-y) [37,38]. However, a study based on typical consumption rates, considering MPs down to 1 µm in size, much smaller than those previously considered, extrapolated much higher maximum human adult uptakes of 458,000 MP/y from tap water and 3,569,000 MP/y from bottled water [39].

It should also be noted that reported MP counts are highly dependent on the sampling protocol adopted in the various studies: [40] highlighted detection methods’ influence on observed MP abundance in freshwater environments around the world, as most published studies limit investigation to MPs in the range 20–5 mm, neglecting lower size particles; however, by pushing experimental protocol below the generally adopted threshold of 20 µm, and even remaining within the MP 1 µm lower limit, up to 2–3 log count detection increases were reported.

### 3.1. MP Detection Issues

A logical question arises at this point: what do MP count numbers actually mean? To answer this, MP definition and detection technologies should be examined. The most astonishing fact is that no legal MP definition existed until very recently (2023), notwithstanding the fact that the European Chemicals Agency (ECHA) had already in 2019 proposed a regulatory definition for MP under REACH (Registration, Evaluation and Authorisation and Restriction of Chemicals) legislation. In that year, the EU unofficially adopted the upper size limit of 5 mm [41]. The first official definition came in 2023, under ISO standard 24187 [42], in which large microplastics are described as any solid plastic particle (insoluble in water) with any dimension between 1 and 5 mm, and microplastic as particles with dimension between 1 and 1000 µm. At the moment, however, other differing definitions, such as those from EU agencies, ISO, and Swedish EPA, complicate data consistency: ECHA defines MPs as particles with dimensions ˂5 mm and fiber particles with lengths ˂15 mm; the Swedish EPA defines MPs as particles with dimensions ranging between 1 nm to 5 mm; finally, the Marine Strategy Framework Directive 2008/56/EC (MSFD) defines MPs as microlitter smaller than 5 mm in the longest dimension and provides no lower limit [43]. In addition to size, types of MPs also need standardization. According to ECHA, MP particles must be organic, insoluble, and resistant to degradation, thus excluding bio-degradable or water-soluble particles above 2 g/L. The Swedish EPA also includes as MPs synthetic and natural rubber (latex) particles as they exhibit similar properties from an environmental perspective. The MSFD uses the term “microlitter”, including metal, paper, and glass particles, which are the most common components of litter found on coastlines; ISO only mentions that particles must be insoluble in water [43].

Following EU Directive 2020/2184 introducing regulations to ensure safe human consumption of drinking water [44], a recent Commission’s Delegated Decision stipulated that MPs must be monitored in drinking water [45]. To support this effort, a Joint Research Center (JRC) report was recently published to standardize MPs in drinking water analytical methods, as sampling techniques significantly affect their detection [46].

A new proposal of the European Council and Parliament concerning urban wastewater treatment (under discussion) indicates that MPs should also be monitored and regulated in wastewater [47]; however, there is still no standardized method for determining MP in wastewater (or sludge). Generally, the same standards for drinking water (e.g., ASTM D8332-20) [48] are also used for wastewater; however, generalization of these procedures required significant modifications due to the challenges originating from much higher content of organic and suspended solids materials in the latter, which may result, in the end, in incomparable data.

Even for different types of natural waters (e.g., from wells, rivers, springs, lakes, estuaries, and oceans, including thawed snow), standardized MP determination methods have not been established yet. For example, the MSFD Technical Group on Marine Litter highlights that the Atlantic Ocean, North Sea, Baltic Sea, and Mediterranean seawaters are being sampled differently, as the trawl’s mesh size, trawling duration, and surface area/length are not standardized. Generally, 45 µm mesh is used for collecting surface water at 1 to 3 knots speed, as smaller (20 µm) mesh is easily clogged by plankton [49]. Thus, most measurements do not include smaller particles. These inconsistencies in MP definition and measurement methods may cause unnecessary challenges when comparing data (e.g., g/L or MP/L) to assess baselines and trends, thresholds, and environmental risk.

Environmental samples are commonly analyzed using either visual analysis (for particles down to ≈50 μm), vibrational spectroscopy, or thermal analysis [43]. Most existing monitoring studies have focused on MP detection by µ-FTIR (micro Fourier-transform infrared spectroscopy) and micro-Raman technologies, whose resolutions are in the order of 20 µm and 1 µm, respectively [50]; both, however, are subject to errors and uncertainty since degraded particles may produce different spectra compared to pristine samples [51]; furthermore, microbiological, organic and inorganic materials can also cause significant interference. For these reasons, only a few studies have successfully identified MPs of 1 μm [52]. Pyrolysis–Gas Chromatography–Mass Spectrometry (Py-GC/MS), on the other hand, can identify polymer type and mass with no lower size limit since the sample is incinerated and analyzed as an entity; however, particle sizing must be made preliminarily either through sieving or filtration. Table 1 summarizes requirements, strengths, and weaknesses of current MP determination methods.

Additionally, as a consequence of the above, most literature on removal technologies refers generically to MPs, either without size specification or often explicitly limited to those >20 µm, due to the complexity of the analytical procedures involved. In some studies, biodegradable MP plastics are excluded [53]. As discussed in the following section, possible implications for human health increase with decreasing particle size; therefore, the World Health Organization (WHO) currently recommends the characterization and quantification of MP in sizes <10 μm and considers current data generally incomplete for proper assessment of human health risk [54]. A more detailed, and still missing, MP classification based on both particle size and material would thus be more appropriate and relevant to the assessment of their effects; in particular, among MPs, nanoplastics (NPs) are one of the least known and characterized pollutants in all environmental media, due to detection and analytical issues.

NPs derive from the continued environmental degradation of MPs but may specifically originate from other potential sources, such as 3-D printer waste, plastic tea bags, and others [55]. Aside from the order-of-magnitude size difference, recent research points to the likelihood that NPs could be far more toxicologically active than MPs, presenting potentially higher hazards to organisms than the latter due to their capability to cross biological barriers [56].

### 3.2. Nanoplatics: An Entirely Separate Issue?

Although there is no consensus on the definition of nanoplastics (NPs), it was initially suggested that these should be defined within the size range of 1 to 1000 nm [57]; Swedish norms include this range in their MP definition, but ISO 24187 does not. On the other hand, both the U.S. National Nanotechnology Initiative [58], the European Commission [59,60,61], and ISO norms [62] define nanoscale materials (engineered nanomaterials) as those having one or more dimensions within the range of 1–100 nm; NPs could logically follow that definition, given that most individual polymer molecules are ˂100 nm in size. Occasionally, the term ‘submicroplastics’ appeared in a few reports describing intermediate particles between 100 and 1000 nm.

To overcome the detection limits (≈1 µm) still affecting sophisticated state-of-the-art technologies [50], new methods for NP detection have been investigated: hyperspectral stimulated Raman scattering (HSRS) microscopy, increasingly used in biomedical imaging, was recently shown to experimentally enable NP detection down to the 100 nm size, differentiating them from other nanoscale nonpolymeric materials [36]. Nano-FTIR [63], Atomic infrared spectroscopy (AFM-IR) [64], Confocal Laser Scanning microscopy (CLSM) [65], and other techniques were recently proposed for NP identification, since they offer significantly higher resolution, reaching down to the 10–20 nm level [63]. In combination with Py-GC/MS, they offer the capability to provide both quantitative and qualitative information on NPs in the environment [63].

Notwithstanding the technical possibility of detecting such small NPs, the technological readiness of these methods could be estimated at TRL 4–5, at most, accessible to a few selected laboratories but still well beyond generalized commercial application. It is, therefore, obvious that the practical feasibility of facile NP detection does not comprise their entire range; secondly, analytical characterization of these particles is challenging for complex matrices since the term “plastics” describes a variety of materials, sometimes with very different properties, and tests may not necessarily recognize them after ‘aging’ due to environmental permanence [66].

The NP issue overlaps with that of nanoscale materials in general (e.g., carbon nanotubes, graphene oxide, titanium dioxide, etc.), which are increasingly used in biomedical, industrial, and environmental applications and face major challenges for experimental quantification even in controlled laboratory conditions [67]. These nanoscale particles may also pose possible threats to human health and the environment [68], similar, considering their physicochemical properties, to those of NPs. It also was postulated that due to their peculiar physical and chemical properties, and environmental and biological fate, NPs should be considered as an entirely different pollutant class rather than be grouped either with MPs or engineered nanomaterials [69,70].

### 3.3. MP/NP Ingestion and Human Health Risk

In the light of current knowledge, a clear distinction between MPs and NPs should therefore be made. For the purpose of this discussion, particles ≥1 µm will be considered MPs, consistent with the approach of the 2022 WHO report [54]. Given the evidence presented, the actual levels of human MP ingestion from drinking water seem highly uncertain, as is the extent of risk represented or implied by mainstream literature [71].

In fact, according to the World Health Organization (WHO), there is still insufficient information to draw definite conclusions on MPs toxicity in humans; while some studies have reported adverse effects, these have substantial limitations, including limited cohort size and insufficient accounting for co-factors; data are also often contradictory, as other studies found no significant correlation between exposure to MPs and claimed adverse effects. Currently, available evidence is therefore considered insufficient to determine whether exposure to MP can be associated directly or indirectly with any pathology. Limited MP hazard characterization suggests that their possible adverse effects may be similar to those of other well-studied solid, insoluble particles through similar acting mechanisms [54].

On the other hand, food-related studies suggest that microparticles <1.5 μm could cross the intestinal epithelium [72], but MPs did not so far show significant bioaccumulation or biomagnification in humans or higher organisms, unlike persistent and toxic pollutants found at concentrations orders of magnitude higher than in the surrounding environment. Recent studies (on 42 hospitalized patients with unrelated diagnoses) estimated the accumulation of MPs in various human tissues to be between 1.40 ± 3.37 and 44.37 ± 91.44 µg/g, predominantly in the lungs, indicating that inhalation seems a prevalent ingestion pathway [73]. When orally ingested, MPs traverse the digestive system, remaining largely unaltered by physical or biochemical agents, including the stomach’s acidic conditions, without substantial alteration of their physicochemical characteristics [74]. Medical studies indicate that MPs in the 50–500 µm range were present in adult stools from different global locations and that those >150 µm are likely to be rapidly excreted in feces, while adsorption of smaller ones is largely unexplored but expected to be limited, increasing with diminishing size [75]. Given the multitude of human exposure pathways to MPs and the possibility of smaller MPs to cross human cell membranes and migrate to different organs, it becomes virtually impossible to ascertain the initial provenance of accumulated MPs in human tissues.

The human risk from MPs ingestion is still unresolved, especially concerning exposure to associated chemicals (due to the so-called ‘vector effect’) related to MPs’ scavenging of dispersed environmental pollutants [76,77,78,79]. A wide spectrum of inorganic and organic pollutants, including PCBs, PFAS, and pharmaceuticals, can, in fact, adsorb on MPs, as well as on NPs [80,81,82]. Potential hazards of MPs are sometimes estimated based on their composition: for example, the vinyl chloride monomer, which carries a high potential risk to human health based on cell studies, accounts for 100% of PVC polymers [83]. Scientifically accurate models should, however, be developed to evaluate MP actual toxicity rather than relying on individual monomers’ toxicity in those molecules since, so far direct cause-effect link between MP ingestion and effects associated with adsorbed contaminants has not been demonstrated yet, and often referred to as ‘complex’, ‘under debate’ or ‘controversial’, by the few studies carried out in environmentally relevant conditions.

Studies on biological human samples for MP determination are subject to high variability, as no standardized procedures exist at the moment, and most such studies are limited to a few repetitions. Toxicological studies on mice, where adverse effects were observed, are of questionable relevance since they were generally conducted with extremely high concentrations and exposure that would not normally occur through drinking water ingestion [84]. Most studies on MP effects on organisms were carried out on small aquatic species at exposure conditions several orders of magnitude higher than those observed in natural environments, usually involving only one or few polymer types and sizes [85].

On the other hand, preliminary in vitro studies on NPs show that, unlike MPs, they could pass through internal biomembranes into the bloodstream and, from there, reach organs, including the heart and brain, enter individual cells, and cross the placenta [74]. Oral administration in mice and cellular studies on human gastric epithelial cells showed that NPs can be absorbed after prolonged exposure [86]; however, information on actual NPs effects in humans is limited and may be assimilated to those of other nonpolymeric nanoparticles. Toxicological concerns of NPs are mostly based on their higher surface/volume ratio and surface reactivity in comparison to larger particles.

Recent studies submitted that the potential MP/NP risk is not dependent solely on concentration but also on polymer type, chemicals eventually absorbed from the environment, and final localization within the human body [83,87,88]. As with MPs, the vector effect of NPs for anthropogenic contaminants and toxic metals has not been thoroughly studied. Much like MPs, and perhaps to a higher degree, NPs have the capacity for organic contaminants adsorption due to their high specific surface area and hydrophobic composition. Additionally, NPs can more easily enter cellular membranes and, thus, may be more effective vectors for contaminants ‘delivery’ within organisms, but this does not necessarily imply that these contaminants would be readily bioavailable: it was shown that low concentrations of polystyrene NPs could actually reduce the concentration and cytotoxicity of phthalate esters on human lung epithelial cells, as a result of phthalate ester being sorbed on NP particles, thus reduced its bioavailability [89].

Plastics contain additives introduced during their production that confer them the desired physical properties; these include softeners, UV stabilizers, flame retardants, and other agents. ECHA lists about 400 such additives, some of which (e.g., phthalates, bisphenols, brominated flame retardants, triclosan, and organotins) are of concern to human health [90]. It was shown that such potentially toxic plastic additives may be gradually released from MP/NP over long periods and may actually bioaccumulate [91]. Such release mechanisms and related toxic effects should be investigated; from preliminary findings, however, it seems likely that slow release of these substances will not occur during the short residence time of larger MPs within human organisms, while release from NPs may be facilitated by their incorporation into biological tissues. As very little is known about the toxicity of MP/NP to humans, more research is needed to systematically address this subject [92,93]. From the evidence available so far, for the purpose of human water consumption exposure effects, attention should be mainly focused on NPs and dissolved compounds, for which traditional drinking water treatment is scarcely effective, as discussed in the following section. More detailed studies on these aspects are hence necessary.

A note is of order on MP/NP human intake from drinking water: as pointed out in several studies [36,94], individuals who mostly drink (plastic) bottled water are likely subject to a much higher lifetime oral particle intake than those consuming mainly tap water. Increased MP (1–5 μm) content in bottled water (partly within the range of possible cell penetration) could derive from the degradation of packaging material: studies showed that MPs released from PET bottles and HDPE caps into the contained water considerably increased after repeated bottle opening and closing cycles [54]. Additionally, these individuals may also be ingesting plastic additives, including bisphenol A, phthalates, alkylphenols, perfluoroalkyl and polyfluoroalkyl substances (PFAS), and organophosphate esters, that can leach into water from the bottles’ material to their content during long-term storage [95].

Although bottled water consumption is virtually an obligatory choice in some regions, due to limited progress or failure of public water supply systems development [96], its exclusive consumption has been associated with increased risk for certain health conditions, with reported detrimental effects on human health. Studies, however, seem to attribute these consequences to leached additives rather than particles themselves; plastic bottled water may contain PPCPs, PFASs, APs, and BPAs at ng/L concentration levels, and phthalates at μg/L levels, showing greater degrees of CEC contamination than glass bottled water [54,97,98,99].

## 4. Drinking Water Treatment Technologies and MP/NP Removal

Studies on MP removal from drinking water showed that particles are removed significantly by coagulation and filtration, with removal efficiency depending on coagulant type, solution chemistry, and polymer type. Up to 56% of MPs may be removed by conventional sand filters; coagulation, flocculation, sedimentation and granular activated carbon (GAC) filtration have shown removal efficiency of 40–54.5% for MP fibres, and 56.8–60.9% for small size MPs [100].

Removal of MPs during coagulation and flocculation processes can be influenced by natural organic matter (NOM) presence, either hindering or promoting the aggregation and settling of MPs, depending on the coagulant type and MP nature [101].

Since literature reporting MP removal efficiencies often does not specify the investigated granulometry, comparison among studies is not immediate, and the variability of reported results is of difficult interpretation. Sand filters are among the most common filtration processes in water purification: in a laboratory sand filtration study, removal efficiencies for 20, 45, and 90 μm MPs varied in the range 77.4–95.3%, with close-to-complete removal of those ≥45 µm in size, but relatively low removal (33.0–41.1%) for those ≤20 μm [102].

Filtration efficiency can be enhanced by pre-coagulation: three conventional (consisting of clariflocculation, sand filtration, chlorination sequence) WTPs in Dhaka (Bangladesh) reduced initial MP (≥20 µm) content of Shitalakshya River’s raw water by more than 98.5% [103]. In various studies, coagulation by Fe- and Al-based salts showed inconsistent efficiency; coagulation/settling with the use of polyacrylamide (PAM) based coagulants resulted in higher (up to 3 fold) MPs counts [78]. Since floc particle size affects collision efficiency and settling behavior, ballasted flocculation (BSF), a physical-chemical separation process employing additives to promote the formation of heavier flocs, with the addition of sand or GAC/PAC [104], could be employed to improve process performance. Electrocoagulation is a relatively cheap treatment process not relying on the reagents used in general chemical coagulation but using metal electrodes to electrically produce them, making the process simple and robust [105]. Electrocoagulation performance for MP removal under laboratory conditions showed removal efficiencies of PE MPs >90% [106].

MP removal by agglomeration-fixation processes using organosilanes [107], as well as other polymers such as alkoxy-silylates [108], were tested: these lead to the formation of larger particles (up to 3-log bigger, easily removed by conventional separation techniques.

A recent study claimed that the surprisingly simple strategy of boiling water can “decontaminate” it from MP/NPs [109]. The study presented evidence that PS, PE, and PP particles can co-precipitate with calcium carbonate (CaCO_3_) in tap water upon boiling; the effect is more pronounced in hard water (>120 mg/L CaCO_3_), in which boiling can remove at least 80% of particles between 0.1–150 μm; in softer water (80 mg/L CaCO_3_), the removal is limited to 4% of particles. The mechanism is reportedly due to high temperature promoting CaCO_3_ nucleation on MP/NPs, resulting in their encapsulation and aggregation within CaCO_3_ precipitate polymorphs (i.e., calcite, aragonite, and vaterite).

Drinking boiled water is an ancient and still persisting tradition in several Asian countries, including China, as it is considered beneficial for human health; in fact, boiling can remove some chemicals and most biological substances. This practice would be highly sustainable as electric kettles to boil water have low energy consumption and thus low CO_2_ emission. After boiling, filtration devices, such as simple fine stainless steel filters (frequently used when preparing tea), are essential to retain CaCO_3_/MP/NP precipitates in prepared water. While not eliminating exposure completely, such universally-implementable low-impact systems could significantly reduce it, especially if previously treated in conventional WTPs.

### 4.1. AOPs and MPs

UV-based processes for disinfection/advanced oxidation in conventional WTPs were shown to induce increased photochemical MP weathering, the release of plastic additives, and related degradation [110,111]. While reporting complete removal of 45–90 µm MPs in sand filters, Na et al. [103] observed breakthrough of those ≤20 µm (1.2% and 16.6% for 20 and 10 µm MPs, respectively), which were then further fragmented by subsequent UV oxidation. Total MP counts increased by 4.1% after UV treatment (6 h) and by 13.2% after UV/H_2_O_2_ treatment, respectively. It should be noted that since MP are generally counted and not weighted (save for the case where Py-GC/MS is used), the same mass of fragmented MP could result in higher counts after certain processes: UV/H_2_O_2_ treatment promotes higher fragmentation and chemical leaching than UV alone.

Likewise, ozonation often results in negative MP removal efficiency, with smaller (1–5 µm) MP counts increasing by 2.8–16.0% after treatment due to their breakdown into smaller particles under combined chemical degradation and the effect of shearing forces. Post-ozonation GAC filtration, however, enhanced removal by 17.2–22.2% [78].

A study at a conventional WTP (flocculation, sand filter, chlorination) in Geneva (Switzerland) reported 70% MP removal (size ≥ 63 µm) after sand filtration and 98% after the addition of ozonation followed by GAC filtration [112]. A comparison of a conventional (coagulation/sedimentation, sand filtration, and chlorination) and an advanced WTP (conventional plus ozonation and GAC filtration) in China showed that the latter removed MPs better (83.0%) than the former (73.3%) [113]. Although this aspect has not been studied extensively, it has been shown that several advanced oxidation processes (AOPs) used for the removal of specific emerging contaminants or disinfection [114] contribute to MP degradation [115,116]. AOPs’ effects on MPs include degradation, decrease of particle size, and mass loss; however, observed polymeric mineralization rates are low, therefore resulting in higher counts of smaller particle generation [117].

### 4.2. Membranes and MPs

Membrane filtration is currently considered the ‘gold standard’ of water and wastewater treatment technology; it works as a physical barrier against all solids and is commonly used for advanced treatment of drinking water due to its high achievable effluent quality [118]. Few specific studies on drinking water MP removal by membrane separation are available since most deal with wastewater applications [38,119].

Comparative studies on MP removal by membranes (0.05 µm porosity) and rapid sand filters in Indonesia showed superior performance of the former by >44% [120]. Membrane filtration can increase MP removal by one order of magnitude, from 2.2 MP/L after primary treatment to 0.28 and 0.21 MP/L after ultrafiltration (UF) and reverse osmosis (RO), respectively [121]; however, negative MP removal efficiency by membrane filtration processes may occur because of polymeric membranes aging and cleaning causing their rupture, thereby increasing the number of MPs in effluents [122]. RO technology, able to retain particles as small as 0.1 nm, is commonly applied for the exploitation of seawater as an alternative supply source in water-scarce areas [123]; due to the very small pore size, MP/NP content in desalinated seawater should be virtually nil. The biggest problem in membrane filtration is fouling, leading to premature degradation of process performance and increasing cost: MP loads in processed water as high as 10^6^–10^7^ MP/day pose an increased risk of fouling, reducing filtration performance and requiring higher process transmembrane pressure (TMP) for operation [124]. Increased TMP increases membrane stress and could potentially induce abrasion or deterioration of the membrane surface, causing polymeric particle dislodgement into the permeate.

### 4.3. NP Removal

Few specific studies have specifically addressed NP removal in WTP due to the discussed detection issues. In a recent study adopting advanced detection methodologies, NP (20–1000 nm) removal efficiency was assessed in a WTP with 10,000 m^3^/d capacity, consisting of conventional coagulation, precipitation, filtration, and disinfection units, supplemented by advanced treatment ozonation and ozone-activated carbon (AC-O_3_) units [74]. Investigated PE and PVC NP counts at different sampling points throughout the WTP train were converted into mass concentration by empirical calibration: in the influent, PE and PVC NPs were detected at 0.86 μg/L and 137.31 μg/L. After ozonation, concentrations increased to 4.49 μg/L and 208.64 μg/L, respectively; a negative removal was also observed in other studies concerning MP ozonation [76,125].

Observed NP concentrations were reduced in the downstream units of an advanced WTP in the Zhejiang Province (China), consisting of a conventional train of coagulation, precipitation, filtration, and disinfection, in which advanced ozonation and ozone-activated carbon filter units were included. Influent and effluent NPs (20–1000 nm) were characterized by AFM-IR followed by Pyr-GC/MS; influent concentrations of PE and PVC NPs were 0.86 μg/L and 137.31 μg/L, respectively, increased to 4.49 μg/L and 208.64 μg/L in the effluent of the ozone contact unit, and further reduced non-detected and 76.83 μg/L PVC NPs in the effluent after GAC filtration [126]. Results indicated that a WTP thus configured could remove NPs to some extent (≈44%); however, process trains must be appropriately studied to achieve that purpose.

Other NP-specific studies reported that their removal in sand filters could be significantly improved (from 58.2% to 99.91%) by reducing flow rates (from 3.6 m/h to 0.48 m/h) [127]. While this is not discussed in the study, it could be hypothesized that lower hydraulic loading rates to the sand filter could favor NPs adsorption onto the bed particles. Studies on engineered nanoparticles, in fact, indicate that they can be removed from water by adsorption [128]. Activated carbon is the most used adsorbent in drinking water treatment due to its properties, practicality, and cost, showing efficiency in the removal of organic and inorganic pollutants [129]. Given the similarities between engineered nanoparticles and NPs, it is logical to hypothesize that the latter could respond well to such a process. Studies showed that NP retention in sand columns could be increased up to ≈98% by the addition of adsorbents (i.e., GAC, Fe_3_O_4_-doped biochar) to the column medium without lowering hydraulic load; removal efficiency of 180 nm PS NPs by anthracite filtration reached 98.9% [130]. A pilot-scale WTP, reproducing at a reduced scale the processes and conditions of a real facility, showed that filtration by sand and GAC mixed filters could achieve an overall NP removal of 88.1% [131].

A study investigating NP (<400 nm) removal from water using conventional filtration, centrifugation, and ballasted flocculation at bench scale showed that filtration (0.22 μm) removed 92 ± 3% of particles without changing their distribution; centrifugation at 10,000 rpm for 10 min removed 99 ± 1% of preferentially larger particles. Ballasted flocculation removed 88 ± 3% of particles [132].

In conclusion, empirical evidence confirms that commonly used processes in WTPs can effectively remove MPs larger than ≈1–10 µm [133]. Limited evidence that smaller-sized NPs, which are scarcely studied and difficult to detect, could also be eliminated by conventional units (e.g., sand filters, GAC) was also presented. In addition, nano- and ultra-filtration and RO membranes could be used for enhanced NP removal thanks to their small pore sizes [134], much the same way that they can effectively remove engineered nanoparticles [135].

Table 2 summarizes the main current technologies for MP/NP removal from drinking water.

At this point, another important question arises: is it actually necessary to implement advanced technologies for MP/NP removal from WTP, and if so, under which circumstances?

## 5. Possible Approches

Several critical issues have emerged from the previous sections. The first concerns experimental protocols variability in MP studies: although the size limits have been recently (2023) set at 1–5 mm for MPs 1 to 1000 µm for NPs [42], a systematic review of published research articles highlighted that the minimum size of particles considered varied from 1 to 100 μm, which is critical when considering reported counts data [36]. Nonstandardized reporting also hinders the reliability and comparability of experimental protocols: as pointed out by a recent systematic review, only one study (out of 12 finally examined according to precise consistency criteria) reported MP counts retrieved from extraction, only four (out of 12) how many particles were analyzed for composition; just seven reported the upper MP size detected [39]. Studies using either FTIR, RM, or SEM-EDX methodologies showed differences in spectral similarity index, number and proportion of particles analyzed, and spectral libraries used. The first rule for obtaining scientifically comparable results is the use of standardized experimental protocols, as in conventional contaminants determinations (e.g., the well-known APHA Standard Methods with reference to conventional parameters) [144]. MP/NP standardized determination methodologies, which should also include size and composition identification, have been long advocated [145], and although it was just recently (partly) developed (with the exclusion of sub-µ particles), it may shed more confusion in the comparison of results from older studies to those of new ones following those protocols.

There is evidence that NP is the most critical particle with regard to removal technologies’ efficiency and to possible human exposure risk, including accumulation in tissues and internal organs, with the possible release of various potentially harmful constituents (metal ions, chemicals). Most MP detection protocols rely on micro-FTIR or micro-Raman methods with resolutions in the order of 20 µm and 1 µm, respectively, which are not suitable, notwithstanding their technological sophistication and complexity, to detect and analyze NPs. This issue concerns not just NPs but also engineered nanomaterial in general, from which the former are of laborious differentiation [146]: a still unanswered scientific question is whether NPs, due to their specific characteristics and behavior, should fall in the MP or in the former category, or should be classified as a contaminant class of their own [69,70].

The second issue concerns the fate of MP/NPs in human organisms, which is different across various tissues and organs due to their different permeability [72,74,147]: studies are needed to assess the potential for accumulation and toxic compounds release of specific sizes and types of particles in the human organism. Experimental laboratory conditions should mirror observable conditions in drinking water treatment and distribution systems (i.e., real-life exposure situations). Particle size distribution in raw and distributed water and their interactions with treatment systems and other environments should be studied to assess the real need for specific MP removal actions in view of their proven toxicity risk. These aspects are essential to assess the need for additional action in WTPs and water distribution systems. Although few studies on MP removal from drinking water include a comprehensive screening of various sizes and compositions, traditional treatments can generally be considered effective in removing the majority of MPs larger than 20 µm, resulting in tap water with ˂2–10 MP/L of that size.

The third issue concerns possible WTP additional treatment needs and the effect of water distribution systems on MP/NP presence due to processes occurring in the pipeline environment itself. Several studies have addressed strategies for remediating MP/NPs from contaminated water. Suggested methods include chemical, biological (bacterial, fungal, enzymatic, including OGM-based), and nanotechnology-based treatments [148]. The drawbacks of most of those technologies are the possibility of further particle breakdown, producing greater, less controllable NP presence, and possible hazardous byproducts resulting from their decomposition [148]. It was previously shown that advanced treatment processes, such as AOPs, may actually increase NP counts by causing MP degradation into smaller particles, possibly increasing the toxic potential of treated water.

Furthermore, studies indicated that transport in water distribution systems may both retain, produce, or release micro- and nano-particles. For example, MP concentrations in WTPs effluents in Germany were observed to exceed those in tap water, indicating that the distribution system may have retained them in pipe scales [34]. Particles in pipe scale samples were found to be smaller than in liquid samples (generally <50 μm, versus up to 100 μm in the latter), possibly due to the stronger adsorption capacity of smaller particles linked to higher specific surface, stronger hydrophobicity, and lower electronegativity [149]. On the other hand, MPs immobilized in pipe scales may be released due to changes in the pipes’ environment, long hydraulic retention times, and shear stress that may lead to MP desorption or abrasion from aging or peeling epoxy paints in cast iron pipes, aging of plastic pipes and fittings, or both [34]. Pipe scales’ MPs may provide growing surfaces for microbes to form, with microbial communities changing over time as a function of hydraulic conditions; opportunistic pathogens, which might be harmful to humans, could also develop and be transported upon particle release [150]. Apart from their occurrence, deposition, and release in the water distribution system, material migration tests indicated that worn elements from WTPs could also be a potential source of MPs [151]. Based on current literature reviews, most MPs can be removed by existing water treatment plants prior to being distributed; however, excess residues in tap water have been detected without a clear major source of contamination after WTP treatment [101]. The dynamics of MP/NPs in distribution system pipes is a still poorly understood phenomenon that may contribute to high final particle counts at household taps [152].

Further, exposure of human targets drinking private well water or relying on public water systems with basic (low treatment) technology has hardly been addressed by past studies. It is highly likely that, even upon a future implementation of the recent Commission’s Delegated Decision on MPs monitoring of drinking water [45], these situations will escape surveillance.

In view of the previous considerations and based on current scientific evidence, it could be concluded that there is no need for additional action for advanced MP removal beyond that demonstrated so far by normally efficient, conventional WTPs. The presence of GAC filtration units in most WTPs greatly improves both MP and NP removal efficiency to varying degrees. In specific situations, should ad hoc investigations highlight critical conditions, and in particular concerning users of untreated supplies, final point-of-use (POU) removal could be implemented, thus also eliminating eventual in-pipe, post-treatment generated MP/NPs. Onsite household filtration of drinking water by consumer POU devices is already popular for the removal of an array of compounds, including heavy metals, fluorides, nitrates, objectionable tastes/odor, and precipitated particulate originating within distribution networks. Commercial POU test studies, generally limited to MPs (>1 µm), showed that commercial devices consisting of different combinations of GAC, ion exchange, microfiltration (0.22 μm), and non-woven membranes demonstrated removal efficiencies greater than 90%, and up to 94.3% [153]. Innovative polyvinyl alcohol (PVA) nanofibrous membranes suitable for POU device applications were recently tested, showing PE MPs (5–25 μm) removal efficiency of 99.7% [154]. Membrane pore sizes >1 µm may still not retain smaller particles: observed retention of 0.1–0.5 µm PS and PMMA particles in POU devices was ˂7% [155].

RO compact domestic systems have been recently put on the market and could be considered an effective way to remove not just MPs but also most NPs at the final POU. One drawback of such systems, curiously, lies in their high particle removal selectivity: the WHO, in fact, recommends against relying solely on RO-treated water for long-term drinking purposes due to its deficiency in trace elements and minerals essential for human health. In Singapore, where RO-treated NEWater is produced from recycled wastewater, providing 30% of the Country’s water needs, the produced water is mainly directed to industrial users for this specific reason [156]. Electrolyte replenishment (remineralization) is needed for long-term drinking uses; while this can be easily performed in a highly controlled fashion in centralized facilities, it could be more problematic and less controllable in small decentralized/domestic systems. Other RO disadvantages may include high maintenance requirements due to filter clogging, potentially increasing in the case of hard water sources, high installation costs, and slow water production in household applications, as the pressure used is generally lower than in industrial facilities. Previous studies also correlated the possibility of increased gastrointestinal disease associated with certain POU RO treatment devices for domestic use [157]. Despite incomplete removal achievable, based on current technological knowledge and maturity, a combination of UF membranes and GAC filtration could be the most efficient one for POU devices.

Future climate scenario uncertainty could affect water availability, highlighting the impending need to build supply resilience and sustainability [158]. The increasing use of marginal water sources may affect the quality of water supplies, and careful evaluation of all health-related parameters will be a growing challenge for the future.

## 6. Conclusions

Micro- and nano-plastic contamination of drinking water resources is a global issue reflecting the pervasive presence of plastics in all environmental media. Oral ingestion is not, in most cases, the prevalent pathway for human exposure, and current scientific evidence does not delineate a generalized situation of ascertained human health hazards due to MP ingestion exposure. The high removal efficiency of many conventional water treatment technologies, in addition, results in limited counts at final points of use. More detailed toxicological evidence is required for an accurate assessment of exposure and potential hazards of orally ingested NPs, particularly concerning additives release and tissue bioaccumulation.

The micro- and nanoplastic diffusion certainly requires careful assessment of applicable scientific standards and potential risks communication, and the need to develop standard approaches to monitor and assess their presence, fate, and impact on water supplies. However, additional MP/NP removal efforts from piped drinking water supplies do not seem, at the moment, justified by actual health impacts and risk evaluation, and in view of the relevance of their contribution to overall human intake of these substances. Simple household methods (e.g., boiling water prior to consumption) and commercial POU technologies capable of high MP removal are already available for application in cases of concern due to high target exposure. However, they are not as effective for the removal of smaller (˂1 µm) NPs. From the limited evidence available so far, NPs may imply greater potential risks than MPs on human health and could, in that case, require the possible introduction of yet commonly unavailable, advanced technologies for their removal from water supplies.

## Figures and Tables

**Table 1:** Requirements, strengths, and weaknesses of current MP determination methods. (modified from [43]).

Method	Strength	Weakness	Notes
Visual analysis	Straightforward. Allows the exam of large filter surfaces, leading to quick analysis.	No polymer identification. Serious risk of particle misidentification.	Requires skilled and experienced analysts. Useful for sample pre-screening prior to other analyses. It can be improved with training and experience.
FTIR	High resolution. Polymer type identification. Less instrument settings than Raman. μ-FTIR: resolution below 20 μm, with automatic sample scan μ-FTIR provides information on MP aging (through carbonyl index).	Measures smaller filter surface area than visual analysis. Commonly used after visual analysis on selected particles, selection bias can occur. Possible fragment counts are overestimated compared to a stereomicroscope. Accuracy affected by MP morphology. It may not identify particles <10 μm. μ-FTIR operation is time-consuming as it measures individual particles (unless using focal plane array-based detection requiring liquid N for cooling.	Advanced instruments require trained personnel and routine maintenance/calibration to operate. Requires cleaner samples: chemical treatment can affect results. Overlapping particles may induce refractive error. Additional costs for special filters (i.e., anodisc, PTFE, gold coated). Spectral libraries affect identification accuracy. Different laboratories use different hit quality indices and spectral matching libraries, resulting in varying matching success. Harmonization of spectral libraries is needed. Expertise in interpreting spectra of weathered particles is essential.
Raman spectroscopy	Higher resolution than visual analysis. Polymer identification. A good complement to visual analysis. Less affected by polymer degradation than FTIR, not affected by thickness. Can identify particle <1 μm. It can be automated to reduce spectral interpretation operating time. μ-Raman in combination with an optical microscope to analyze particles ˂1 μm.	Risk of contamination by adhesive polymer fragments on instrument surface. Spectra interfered by particle color, addictive, fluorescence, and pigment content. Risk of sample damage by laser beam	Advanced instruments require trained personnel and routine maintenance/calibration to operate. Requires clean sample to reduce spectral interference. Similar to FTIR, different spectral libraries influence final results.
Py-GC/MS	Identifies the total mass of each polymer type in a sample. Characterization of both polymers and additives	No size class of particles is given unless prior particles are manual sorting.	Advanced instruments require trained personnel and routine maintenance/calibration to operate. Requires a clean sample to achieve a cleaner program. Requires dedicated libraries for polymers and additives.

**Table 2:** Summary of main technologies for the removal of MP/NPs in water.

Approach	Advantages	Disadvantages	Principle	MPs Type	MPs Size	Removal Efficiency	Refs.
Membrane Filtration	High removal efficiency	Membrane fouling, High TMP required	UF, RO RO	All All	1–5000 μm 20–1000 nm	≈100% up to 99%	[124,125,136]
Sand filtration	Effective for larger-size MPs	Low removal efficiency	Rapid sand filtration	All	<10 μm	29.0–44.4%	[100]
	Effective for small size particles	Removal efficiency can be improved by adsorbents addition	Low rate filtration	All	20–1000 nm	Up to 99%	[125]
Adsorption	High efficiency, simple operation	Adsorbent regeneration	GAC	All	20–1000 nm	Up to 99.9%	[130]
			Zn/Al layered hydroxides	PS	55 nm	96%	[137]
			Metal–organic framework- foams	PS, PMMA, PVDF	325 nm, 183 nm, 260 nm	88.2% 85.7% 90.1%	[138]
Magnetic removal	Simple, economical, and fast	Addition of magnetic materials to treated solution	MagPOM–SILP	PS	1 or 10 μm	100%	[139]
			Nano-Fe_3_O_4_	PE, PP, PS, PET	200–900 μm	62.83–86.87%	[140]
			M−CNTs	PA, PET, PE	48 μm	100%	[141]
Coagulation	Simple operation, low cost	Consumption of flocculants	Coagulation	PS, PE	<5000 μm	77.83%, 29.70%	[142]
			Coagulation	PE	<5000 μm	8.3–61.2%	[124]
			Coagulation	All MP	<5000 μm	40.5–54.5%	[100]
			Coagulation + GAC	All MP	<5000 μm	62%	[143]
Co-precipitation	Simple requires water boiling	Only effective in hard water	CaCO_3_ precipitation	PS, PE, PP			[109]

## Data Availability

No new data were generated in this study.

## References

[1] ENTECH The Value of Plastics. https://www.entecpolymers.com/resources/product-guides/the-value-of-plastics#:~:text=Plastics%20require%202%25%20to%204,generate%2061%25%20more%20greenhouse%20gases!.

[2] Meng F., Brandão M., Cullen J.M. (2024). Replacing Plastics with Alternatives Is Worse for Greenhouse Gas Emissions in Most Cases. Environ. Sci. Technol..

[3] McKinsey & Company Climate Impact of Plastics. https://www.mckinsey.com/industries/chemicals/our-insights/climate-impact-of-plastics.

[4] Sustainable Packaging Coalition 2021–2022 Centralized Study on the Availability of Recycling. https://sustainablepackaging.org/wp-content/uploads/2022/03/UPDATED-2020-21-Centralized-Study-on-Availability-of-Recycling-SPC-3-2022.pdf.

[5] d’Ambrières W. (2019). Plastics recycling worldwide: Current overview and desirable changes. Field Actions Sci. Rep..

[6] Capodaglio A.G. (2024). Developments and Issues in Renewable Ecofuels and Feedstocks. Energies.

[7] Geyer R., Letcher T.M. (2020). Chapter 2—Production. Production, Use and Fate of Synthetic Polymers in Plastic Waste and Recycling.

[8] Boucher J., Friot D. (2017). Primary Microplastics in the Oceans: A Global Evaluation of Sources.

[9] UNEP (2021). Drowning in Plastics—Marine Litter and Plastic Waste Vital Graphics.

[10] Gewert B., Plassmann M.M., MacLeod M. (2015). Pathways for degradation of plastic polymers floating in the marine environment. Environ. Sci. Process Imp..

[11] White J.R. (2006). Polymer ageing: Physics, chemistry or engineering? Time to reflect. Compt. Rend. Chim..

[12] Burns E.E., Boxall A.B.A. (2018). Microplastics in the aquatic environment: Evidence for or against adverse impacts and major knowledge gaps. Environ. Toxicol. Chem..

[13] Aves A.R., Revell L.E., Gaw S., Ruffell H., Schuddeboom A., Wotherspoon N.E., LaRue M., McDonald A.J. (2022). First evidence of microplastics in Antarctic snow. Cryosphere.

[14] Bank M.S., Hansson S.V., Bank M.S. (2022). Chapter 1—The Microplastic Cycle: An Introduction to a Complex Issue. Microplastic in the Environment: Pattern and Process.

[15] PLASTICS FOR CHANGE The 7 Different Types of Plastic. https://www.plasticsforchange.org/blog/different-types-of-plastic.

[16] (2019). Our World in Data. Mismanaged Plastic Waste. https://ourworldindata.org/grapher/plastic-waste-mismanaged.

[17] Capodaglio A.G. (2024). Microplastics in the urban water cycle: A critical analysis of issues and of possible (needed?) solutions. Sci. Total Environ..

[18] Choudhury T.R., Riad S., Uddin F.J., Maksud M.A., Alam M.A., Chowdhury A.M.S., Mubin A.L., Towfiqul Islam A.R.M., Malafaia G. (2024). Microplastics in multi-environmental compartments: Research advances, media, and global management scenarios. J. Contam. Hydrol..

[19] Travis C.C., Hester S.T. (1991). Global chemical pollution. Environ. Sci. Technol..

[20] Copetti D., Marziali L., Viviano G., Valsecchi L., Guzzella L., Capodaglio A.G., Tartari G., Polesello S., Valsecchi S., Mezzanotte V. (2019). Intensive monitoring of conventional and surrogate quality parameters in a highly urbanized river affected by multiple combined sewer overflows. Water Sci. Technol. Water Supply.

[21] Xia W., Rao Q., Deng X., Chen J., Xie P. (2020). Rainfall is a significant environmental factor of microplastic pollution in inland waters. Sci. Total Environ..

[22] Allen S., Allen D., Moss K., Le Roux G., Phoenix V.R., Sonke J.E. (2020). Examination of the ocean as a source for atmospheric microplastics. PLoS ONE.

[23] The New York Times The ‘Great Pacific Garbage Patch’ Is Ballooning, 87,000,000,000 Tons of Plastic and Counting. https://www.nytimes.com/2018/03/22/climate/great-pacific-garbage-patch.html#:~:text=In%20the%20Pacific%20Ocean%20between,’%20worth%2C%20researchers%20said%20Thursday.

[24] Agostini L., Fornazier Moreira J.C., Gonçalves Bendia A., Pezzo Kmit M.C., Waters L.G., Ferreira Mourão Santana M., Sumida M.Y.G., Turra A., Pellizari V.H. (2021). Deep-sea plastisphere: Long-term colonization by plastic-associated bacterial and archaeal communities in the Southwest Atlantic Ocean. Sci. Total Environ..

[25] Vaksmaa A., Vielfaure H., Polerecky L., Kienhuis M.V.M., van der Meer M.T.J., Pflüger T., Egger M., Niemann H. (2024). Biodegradation of polyethylene by the marine fungus Parengyodontium album. Sci. Total Environ..

[26] Jebashalomi V., Partheeban E.C., Rajaram R. (2024). Microbial degradation of low-density polyethylene (LDPE) and polystyrene using Bacillus cereus (OR268710) isolated from plastic-polluted tropical coastal environment. Sci. Total Environ..

[27] Mishra S., Dash D., Das A.P. (2024). Aquatic Microbial Diversity on Plastisphere: Colonization and Potential Role in Microplastic Biodegradation. Geomicrobiol. J..

[28] Philp R.B. (2013). Ecosystems and Human Health: Toxicology and Environmental Hazards.

[29] Zhao S., Zhu L., Wang T., Li D. (2014). Suspended microplastics in the surface water of the Yangtze estuary system, China: First observations on occurrence, distribution. Marine Poll. Bull..

[30] Tulcan R.X.S., Lu X. (2024). Microplastics in ports worldwide: Environmental concerns or overestimated pollution levels?. Crit. Rev. Environ. Sci. Technol..

[31] Schell T., Rico A., Vighi M. (2020). Occurrence, fate and fluxes of plastics and microplastics in terrestrial and freshwater ecosystems. Rev. Environ. Contam. Toxicol..

[32] Bäuerlein P.S., Hofman-Caris R.C.H.M., Pieke E.N., ter Laak T.L. (2022). Fate of microplastics in the drinking water production. Water Res..

[33] Sangkham S., Islam M.A., Adhikari S., Kumar R., Sharma P., Sakunkoo P., Bhattacharya P., Tiwari A. (2023). Evidence of microplastics in groundwater: A growing risk for human health. Groundwater Sustain. Dev..

[34] Mintenig S.M., Löder M.G.J., Primpke S., Gerdts G. (2019). Low numbers of microplastics detected in drinking water from ground water sources. Sci. Total Environ..

[35] Kosuth M., Mason S.A., Wattenberg E.V. (2018). Anthropogenic contamination of tap water, beer, and sea salt. PLoS ONE.

[36] Qian N., Gao X., Lang X., Deng H., Bratu T.M., Chen Q., Stapleton P., Yan B., Min W. (2024). Rapid single-particle chemical imaging of nanoplastics by SRS microscopy. Proc. Natl. Acad. Sci. USA.

[37] Cox K.D., Covernton G.A., Davies H.L., Dower J.F., Juanes F., Dudas S.E. (2019). Human Consumption of Microplastics. Environ. Sci. Technol..

[38] Shao L., Li Y., Jones T., Santosh M., Liu P., Zhang M., Xu L., Li W., Lu J., Yang C.X. (2022). Airborne microplastics: A review of current perspectives and environmental implications. J. Clean. Prod..

[39] Danopoulos E., Twiddy M., Rotchell J.M. (2020). Microplastic contamination of drinking water: A systematic review. PLoS ONE.

[40] Shen M., Song B., Zhu Y., Zeng G., Zhang Y., Yang Y., Wen X., Chen M., Yi H. (2020). Removal of microplastics via drinking water treatment: Current knowledge and future directions. Chemosphere.

[41] Bujnicki J., Dykstra P., Fortunato E., Grobert N., Heuer R., Keskitalo C., Nurse P. Environmental and Health Risks of Microplastic Pollution, Scientific Opinion 6/2019; Directorate-General for Research and Innovation, European Commission, Group of Chief Scientific Advisors. Brussels 2019. https://op.europa.eu/en/publication-detail/-/publication/f235d1e3-7c4d-11e9-9f05-01aa75ed71a1/language-en.

[42] (2023). Principles for the Analysis of Microplastics Present in the Environment.

[43] Chen C.Y., Olshammar M., Thorsén G., Strömberg E. (2024). Identification and Quantification Techniques for Microplastics: Strengths, Weaknesses, and Recommendations for Harmonization.

[44] EU (2020). Directive 2020/2184 of the European Parliament and of the Council of 16 December 2020 on the quality of water intended for human consumption. Off. J. Eur. Union L.

[45] EU (2024). Commission Delegated Decision 2024/1441 of 11 March 2024 supplementing Directive (EU) 2020/2184 of the European Parliament and of the Council by laying down a methodology to measure microplastics in water intended for human consumption (notified under document C(2024) 1459). Off. J. Eur. Union L Ser..

[46] Belz S., Cella C., Geiss O., Gilliland D., La Spina R., Mėhn D., Sokull-Kluettgen B. (2024). Analytical Methods to Measure Microplastics in Drinking Water.

[47] European Commission (2022). Proposal for a Directive of the European Parliament and of the Council Concerning Urban Wastewater Treatment.

[48] (2020). Standard Practice for Collection of Water Samples with High, Medium, or Low Suspended Solids for Identification and Quantification of Microplastic Particles and Fibers.

[49] Galgani F., Ruiz Orejon Sanchez Pastor L., Ronchi F., Tallec K., Fischer E., Matiddi M., Anastasopoulou A., Andresmaa E., Angiolillo M., Bakker Paiva M. (2023). Guidance on the Monitoring of Marine Litter in European Seas.

[50] Sobhani Z., Al Amin M., Naidu R., Megharaj M., Fang C. (2019). Identification and visualisation of microplastics by Raman mapping. Anal. Chim. Acta.

[51] Dong M., Zhang Q., Xing X., Chen W., She Z., Luo Z. (2020). Raman spectra and surface changes of microplastics weathered under natural environments. Sci. Total Environ..

[52] Xu J.L., Thomas K.V., Luo Z., Gowen A.A. (2019). FTIR and Raman imaging for microplastics analysis: State of the art, challenges and prospects. TrAC Trends Anal. Chem..

[53] RIVM (2019). Factsheet over Microplastics in Nederlandse Wateren. https://www.rivm.nl/sites/default/files/2019-06/Factsheet%20Microplastics%20in%20Nederlandse%20wateren.pdf.

[54] WHO (2022). Dietary and Inhalation Exposure to Nano- and Microplastic Particles and Potential Implications for Human Health. Nutrition and Food Safety (NFS), Standards & Scientific Advice on Food Nutrition (SSA).

[55] Mortensen N.P., Johnson L.H., Grieger K.D., Ambroso J.L., Fennell T.R. (2019). Biological interactions between nanomaterials and placental development and function following oral exposure. Reprod. Toxicol..

[56] Hughes M.F., Clapper H.M., Burgess R.T., Ho K.T. (2021). Human and ecological health effects of nanoplastics: May not be a tiny problem. Curr. Opin. Toxicol..

[57] Gigault L., Halle A.T., Baudrimont M., Pascal P.Y., Gauffre F., Phi T.L., El Hadri H., Grassl B., Reynaud S. (2018). Current opinion: What is a nanoplastic?. Environ. Pollut..

[58] (2018). N.N.I. Nanotechnology—Big Things from a Tiny World.

[59] EC COMMISSION RECOMMENDATION on the Definition of Nanomaterial. http://eur-lex.europa.eu/LexUreServ/LexUriServ.do?uri=OJ:L:2011:275:0038:0040:EN:PDF.

[60] EC (2009). Regulation (EC) No 1223/2009 of the European Parliament and of the Council of 30 November 2009 on cosmetic products (recast). Off. J. Eur. Union L.

[61] EU (2015). Regulation (EU) 2015/2283 of the European Parliament and of the Council of 25 November 2015 on novel foods, amending Regulation (EU) No 1169/2011 of the European Parliament and of the Council and repealing Regulation (EC) No 258/97 of the European Parliament and of the Council and Commission Regulation (EC) No 1852/2001. Off. J. Eur. Union L.

[62] (2023). Nanotechnologies—Vocabulary—Part 1: Core Vocabulary.

[63] Enyoh C.E., Wang Q., Chowdhury T., Wang W., Lu S., Xiao K., Chowdhury M.A.H. (2021). New Analytical Approaches for Effective Quantification and Identification of Nanoplastics in Environmental Samples. Processes.

[64] Xie D., Fang H., Zhao X., Lin X., Su Z. (2025). Identification of microplastics and nanoplastics in environmental water by AFM-IR. Anal. Chim. Acta.

[65] Morgana S., Casentini B., Tirelli V., Grasso F., Amalfitano S. (2024). Fluorescence-based detection: A review of current and emerging techniques to unveil micro/ nanoplastics in environmental samples. TrAC Trends Analyt Chem..

[66] Hurley R.R., Nizzetto L. (2018). Fate and occurrence of micro(nano)plastics in soils: Knowledge gaps and possible risks. Curr. Opin. Environ. Sci. Health.

[67] Vladitsi M., Nikolaou C., Kalogiouri N.P., Samanidou V.F. (2022). Analytical Methods for Nanomaterial Determination in Biological Matrices. Methods Protoc..

[68] Kumah E.A., Fopa R.D., Harati S., Boadu P., Zohoori F.V., Pak T. (2023). Human and environmental impacts of nanoparticles: A scoping review of the current literature. BMC Publ. Health.

[69] Gigault J., El Hadri H., Nguyen B., Grassi B., Rowenczyk L., Tufenkji N., Fent S., Wiesner M. (2021). Nanoplastics are neither microplastics nor engineered nanoparticles. Nat. Nanotechnol..

[70] Chen Z., Shi X., Zhang X., Wu L., Wei W., Ni B.J. (2023). Nanoplastics are significantly different from microplastics in urban waters. Water Res. X.

[71] Burton G.A. (2017). Stressor Exposures Determine Risk: So, Why Do Fellow Scientists Continue To Focus on Superficial Microplastics Risk?. Environ. Sci. Technol..

[72] European Food Safety Authority (2016). Presence of microplastics and nanoplastics in food, with particular focus on seafood. Panel on Contaminants in the Food Chain. EFSA J..

[73] Zhu L., Kang Y.L., Ma M.D., Wu Z.X., Zhang L., Hu R.X., Xu Q., Zhu J., Gu X., An L. (2024). Tissue accumulation of microplastics and potential health risks in human. Sci. Total Environ..

[74] Li Y., Chen L., Zhou N., Chen Y., Ling Z., Xiang P. (2024). Microplastics in the human body: A comprehensive review of exposure, distribution, migration mechanisms, and toxicity. Sci. Total Environ..

[75] Schwabl P., Koppel S., Konigshofer P., Bucsics T., Trauner M., Reiberger T., Liebmann B. (2019). Detection of various microplastics in human stool. A prospective case series. Ann. Intern. Med..

[76] Adeleye A.T., Bahar M.M., Megharaj M., Fang C., Rahman M.M. (2024). The Unseen Threat of the Synergistic Effects of Microplastics and Heavy Metals in Aquatic Environments: A Critical Review. Curr. Poll. Rep..

[77] Barhoumi B., Sander S.G., Tolosa I. (2022). A review on per- and polyfluorinated alkyl substances (PFASs) in microplastic and food-contact materials. Environ. Res..

[78] Wang T., Wang L., Chen Q., Kalogerakis N., Ji R., Ma Y. (2020). Interactions between microplastics and organic pollutants: Effects on toxicity, bioaccumulation, degradation, and transport. Sci. Total Environ..

[79] Kinigopoulou V., Pashalidis I., Kalderis D., Anastopoulos I. (2022). Microplastics as carriers of inorganic and organic contaminants in the environment: A review of recent progress. J. Mol. Liquids.

[80] Puckowski A., Cwięk W., Mioduszewska K., Stepnowski P., Białk-Bielińska A. (2021). Sorption of pharmaceuticals on the surface of microplastics. Chemosphere.

[81] Torres F.G., Dioses-Salinas D.C., Pizarro-Ortega C.I., De-la-Torre G.E. (2021). Sorption of chemical contaminants on degradable and non-degradable microplastics: Recent progress and research trends. Sci. Total Environ..

[82] Nguyen M.K., Rakib M.R.J., Lin C., Hung N.T.Q., Le V.G., Nguyen H.L., Malafaia G., Idris A.M. (2023). A comprehensive review on ecological effects of microplastic pollution: An interaction with pollutants in the ecosystems and future perspectives. Trends Anal. Chem..

[83] Danopoulos E., Twiddy M., West R., Rotchell J.M. (2022). A rapid review and meta-regression analyses of the toxicological impacts of microplastic exposure in human cells. J. Hazard. Mater..

[84] Backhaus T., Wagner M. (2020). Microplastics in the Environment: Much Ado about Nothing? A Debate. Glob. Chall..

[85] Phuong N.N., Zalouk-Vergnoux A., Poirier L., Kamari A., Châtel A., Mouneyrac C., Lagarde F. (2016). Is there any consistency between the microplastics found in the field and those used in laboratory experiments?. Environ. Pollut..

[86] Domenech J., Hernandez A., Rubio L., Marcos R., Cortes C. (2020). Interactions of polystyrene nanoplastics with in vitro models of the human intestinal barrier. Arch. Toxicol..

[87] Wright S.L., Kelly F.J. (2017). Plastic and Human Health: A Micro Issue?. Environ. Sci. Technol..

[88] Ferguson L., Awe A., Sparks C. (2024). Microplastic concentrations and risk assessment in water, sediment and invertebrates from Simon’s Town, South Africa. Heliyon.

[89] Shi Q., Tang J., Wang L., Liu R., Giesy J.P. (2021). Combined cytotoxicity of polystyrene nanoplastics and phthalate esters on human lung epithelial A549 cells and its mechanism. Ecotoxicol. Environ. Saf..

[90] ECHA Plastic Additives Initiative. https://echa.europa.eu/mapping-exercise-plastic-additives-initiative.

[91] Yu Y., Kumar M., Bolan S., Padhye L.P., Bolan N., Li S., Wang L., Hou D., Li Y. (2024). Various additive release from microplastics and their toxicity in aquatic environments. Environ. Pollut..

[92] Barceló D., Picó Y., Alfarhan A.H. (2023). Microplastics: Detection in human samples, cell line studies, and health impacts. Environ. Toxicol. Pharmacol..

[93] Paul M.B., Stock V., Cara-Carmona J., Lisicki E., Shopova S., Fessard V., Braeuning A., Sieg H., Bohmert L. (2020). Micro- and nanoplastics—Current state of knowledge with the focus on oral uptake and toxicity. Nanoscale Adv..

[94] Vega-Herrera A., Garcia-Tornè M., Borrell-Diaz X., Abad E., Llorca M., Villanueva C.M., Farré M. (2023). Exposure to micro(nano)plastics polymers in water stored in single-use plastic bottles. Chemosphere.

[95] Becerra-Herrera M., Arismendi D., Molina-Balmaceda A., Uslar J., Manzo V., Richter P., Caraballo M.A. (2022). Initial phthalates fingerprint and hydrochemical signature as key factors controlling phthalates concentration trends in PET-bottled waters during long storage times. Food Chem..

[96] UNU Bottled Water Masks World’s Failure to Supply Safe Water for All. United Nations University. https://unu.edu/press-release/bottled-water-masks-worlds-failure-supply-safe-water-all.

[97] Akhbarizadeh R., Dobaradaran S., Schmidt T.C., Nabipour I., Spitz J. (2020). Worldwide bottled water occurrence of emerging contaminants: A review of the recent scientific literature. J. Hazard. Mater..

[98] Bach C., Dauchy X., Chagnon M.C., Etienne S. (2012). Chemical compounds and toxicological assessments of drinking water stored in polyethylene terephthalate (PET) bottles: A source of controversy reviewed. Water Res..

[99] Dolcini J., Chiavarini M., Firmani G., Ponzio E., D’Errico M.M., Barbadoro P. (2024). Consumption of Bottled Water and Chronic Diseases: A Nationwide Cross-Sectional Study. Int. J. Environ. Res. Public Health.

[100] Wang Z., Lin T., Chen W. (2020). Occurrence and removal of microplastics in an advanced drinking water treatment plant (ADWTP). Sci. Total Environ..

[101] Romphophak P., Faikhaw O., Sairiam S., Thuptimdang P., Coufort-Saudejaud C. (2024). Removal of microplastics and nanoplastics in water treatment processes: A systematic literature review. J. Water Process Eng..

[102] Na S.H., Kim M.J., Kim J.T.Y., Jeong S., Lee S., Chung J., Kim E.U. (2021). Microplastic removal in conventional drinking water treatment processes: Performance, mechanism, and potential risk. Water Res..

[103] Islam M.S., Islam Z., Jamal A.H.M.S.I.M., Momtaz N., Beauty S.A. (2023). Removal efficiencies of microplastics of the three largest drinking water treatment plants in Bangladesh. Sci. Total Environ..

[104] Callegari A., Boguniewicz-Zablocka J., Capodaglio A.G. (2017). Experimental application of an advanced separation process for NOM removal from surface drinking water supply. Separations.

[105] Garcia-Segura S., Eiband M.M.S.G., de Melo J.V., Martinez-Huitle C.A. (2017). Electrocoagulation and advanced electrocoagulation processes: A general review about the fundamentals, emerging applications and its association with other technologies. J. Electroanal. Chem..

[106] Perren W., Wojtasik A., Cai Q. (2018). Removal of microbeads from wastewater using electrocoagulation. ACS Omega.

[107] Sturm M.T., Horn H., Schuhen K. (2021). Removal of microplastics from waters through agglomeration-fixation using organosilanes—Effects of polymer types, water composition and temperature. Water.

[108] Herbort A.F., Sturm M.T., Fiedler S., Abkai G., Schuhen K. (2018). Alkoxy-silyl induced agglomeration: A new approach for the sustainable removal of microplastic from aquatic systems. J. Polym. Environ..

[109] Yu Z., Wang J.J., Liu L.Y., Li Z., Zeng E.Y. (2024). Drinking Boiled Tap Water Reduces Human Intake of Nanoplastics and Microplastics. Environ. Sci. Technol. Lett..

[110] Song Y.K., Hong S.H., Jang M., Han G.M., Jung S.W., Shim W.J. (2017). Combined Effects of UV Exposure Duration and Mechanical Abrasion on Microplastic Fragmentation by Polymer Type. Environ. Sci. Technol..

[111] Chen C., Chen L., Yao Y., Artigas F., Huang Q., Zhang W. (2019). Organotin Release from Polyvinyl Chloride Microplastics and Concurrent Photodegradation in Water: Impacts from Salinity, Dissolved Organic Matter, and Light Exposure. Environ. Sci. Technol..

[112] Velasco A.N., Ramseier Gentile S., Zimmermann S., Le Coustumer P., Stoll S. (2023). Contamination and removal efficiency of microplastics and synthetic fibres in a conventional drinking water treatment plant in Geneva, Switzerland. Sci. Total Environ..

[113] Han Z., Jiang J., Xia J., Yan C., Cui C. (2024). Occurrence and fate of microplastics from a water source to two different drinking water treatment plants in a megacity in eastern China. Environ. Pollut..

[114] Capodaglio A.G. (2017). High-energy oxidation process: An efficient alternative for wastewater organic contaminants removal. Clean. Technol. Environ. Policy.

[115] de Oliveira Dos Santos N., Busquets R., Campos L.C. (2023). Insights into the removal of microplastics and microfibres by Advanced Oxidation Processes. Sci. Total Environ..

[116] Luo H., Zeng Y., Zhao Y., Xiang Y., Li Y., Pan X. (2021). Effects of advanced oxidation processes on leachates and properties of microplastics. J. Hazard. Mater..

[117] Wang X., Dai Y., Li Y., Yin L. (2024). Application of advanced oxidation processes for the removal of micro/nanoplastics from water: A review. Chemosphere.

[118] Capodaglio A.G. (2020). Fit-for-purpose urban wastewater reuse: Analysis of issues and available technologies for sustainable multiple barrier approaches. Crit. Rev. Environ. Sci. Technol..

[119] Acarer S. (2023). A review of microplastic removal from water and wastewater by membrane technologies. Water Sci. Technol..

[120] Marsono B.D., Yuniarto A., Purnomo A., Soedjono E.S. (2022). Comparison performances of microfiltration and rapid sand filter operated in water treatment plant. OIP Conf. Ser. Earth Environ. Sci..

[121] Ziajahromi S., Neale P.A., Rintoul L., Leusch F.D. (2017). Wastewater treatment plants as a pathway for microplastics: Development of a new approach to sample wastewater-based microplastics. Water Res..

[122] Ding H., Zhang J., He H., Zhu Y., Dionysiou D.D., Liu Z., Zhao C. (2021). Do membrane filtration systems in drinking water treatment plants release nano/microplastics?. Sci. Total Environ..

[123] Angelakis A.N., Tchobanoglous G., Capodaglio A.G., Tzanakakis V.A. (2024). The Importance of Nonconventional Water Resources under Water Scarcity. Water.

[124] Ma B., Xue W., Hu C., Liu H., Qu J., Li L. (2019). Characteristics of microplastic removal via coagulation and ultrafiltration during drinking water treatment. Chem. Eng. J..

[125] Pulido-Reyes G., Magherini L., Bianco C., Sethi R., von Gunten U., Kaegi R., Mitrano D.M. (2022). Nanoplastics removal during drinking water treatment: Laboratory- and pilot-scale experiments and modelling. J. Hazard. Mater..

[126] Li Y., Zhang C., Tian Z., Cai X., Guan B. (2024). Identification and quantification of nanoplastics (20–1000 nm) in a drinking water treatment plant using AFM-IR and Pyr-GC/MS. J. Hazard. Mater..

[127] Inyang M., Gao B., Wu L., Yao Y., Zhang M., Liu L. (2013). Filtration of engineered nanoparticles in carbon-based fixed bed columns. Chem. Eng. J..

[128] Piplai T., Kumar A., Alappat B.J. (2017). Removal of mixture of ZnO and CuO nanoparticles (NPs) from water using activated carbon in batch kinetic studies. Water Sci. Technol..

[129] Tong H., He L., Rong H., Li M., Kim H. (2020). Transport behaviors of plastic particles in saturated quartz sand without and with biochar/Fe3O4-biochar amendment. Water Res..

[130] Zhang Y., Diehl A., Lewandowski A., Gopalakrishnan K., Baker T. (2020). Removal efficiency of micro- and nanoplastics (180 nm–125 μm) during drinking water treatment. Sci. Total Environ..

[131] Ramirez Arenas L., Gentile S.R., Zimmermann S., Stoll S. (2022). Fate and removal efficiency of polystyrene nanoplastics in a pilot drinking water treatment plant. Sci. Total Environ..

[132] Murray A., Örmeci B. (2020). Removal Effectiveness of Nanoplastics (<400 nm) with Separation Processes Used for Water and Wastewater Treatment. Water.

[133] Pivokonsky M., Cermakova L., Novotna K., Peer P., Cajthaml T., Janda V. (2018). Occurrence of microplastics in raw and treated drinking water. Sci. Total Environ..

[134] Keerthana Devi M., Karmegam N., Manikandan S., Subbaiya R., Song H., Kwon E.E., Sarkar B., Bolan N., Kim W., Rinklebe J. (2022). Removal of nanoplastics in water treatment processes: A review. Sci. Total Environ..

[135] Li J., Mubashar M., Zulekha R., Xu C., Zhang X. (2025). Applications of coagulation-sedimentation and ultrafiltration for the removal of nanoparticles from water. Sep. Purif. Technol..

[136] Li J., Wang B., Chen Z., Ma B., Chen J.P. (2021). Ultrafiltration membrane fouling by microplastics with raw water: Behaviors and alleviation methods. Chem. Eng. J..

[137] Tiwari E., Singh N., Khandelwal N., Monikh F.A., Darbha G.K. (2020). Application of Zn/Al layered double hydroxides for the removal of nano-scale plastic debris from aqueous systems. J. Hazard. Mater..

[138] Chen Y., Chen Y., Miao C., Wang Y., Gao G., Yang R., Zhu H., Wang J., Li S., Lan Y. (2020). Metal–organic framework-based foams for efficient microplastics removal. J. Mater. Chem. A.

[139] Misra A., Zambrzycki C., Kloker G., Kotyrba A., Anjass M.H., Castillo J.F., Mitchell S.G., Guettel R., Streb C. (2020). Water Purification and Microplastics Removal using Magnetic Polyoxometalate-Supported Ionic Liquid Phases (magPOM-SILPs). Angew. Chem. Int. Ed..

[140] Shi X., Zhang X., Gao W., Zhang Y., He D. (2022). Removal of microplastics from water by magnetic nano-Fe3O4. Sci. Total Environ..

[141] Tang Y., Zhang S., Su Y., Wu D., Zhao Y., Xie B. (2021). Removal of microplastics from aqueous solutions by magnetic carbon nanotubes. Chem. Eng. J..

[142] Zhou G., Wang Q., Li J., Li Q., Xu H., Ye Q., Wang Y., Shu S., Zhang J. (2021). Removal of polystyrene and polyethylene microplastics using PAC and FeCl3 coagulation: Performance and mechanism. Sci. Total Environ..

[143] Pivokonsky M., Pivokonska L., Novotna K., Cermakova M., Klimtova M. (2020). Occurrence and fate of microplastics at two different drinking water treatment plants within a river catchment. Sci. Total Environ..

[144] Lipps W.C., Braun-Howland E.B., Baxter T.E., APHA, AWWA, WEF (2023). Standard Methods for the Examination of Water and Wastewater.

[145] Twiss M.R. (2016). Standardized methods are required to assess and manage microplastic contamination of the Great Lakes system. J. Great Lakes Res..

[146] Jiang C., Liu S., Zhang T., Liu Q., Alvarez P.J.J., Chen W. (2022). Current Methods and Prospects for Analysis and Characterization of Nanomaterials in the Environment. Environ. Sci. Technol..

[147] Ribeiro F., O’Brien J.W., Galloway T., Thomas K.V. (2019). Accumulation and fate of nano- and micro-plastics and associated contaminants in organisms. TrAC Trends Anal. Chem..

[148] Tripathi M., Singh P., Pathak S., Manimekalai R., Garg D., Dashora K. (2025). Strategies for the Remediation of Micro- and Nanoplastics from Contaminated Food and Water: Advancements and Challenges. J. Xenobiot..

[149] Ma J., Zhao J., Zhu Z., Li L., Yu F. (2019). Effect of microplastic size on the adsorption behavior and mechanism of triclosan on polyvinyl chloride. Environ. Pollut..

[150] Chen X., Lian X.-Y., Wang Y., Chen S., Sun Y.-R., Tao G.-L., Tan Q.-W., Feng J.-C. (2023). Impacts of hydraulic conditions on microplastics biofilm development, shear stresses distribution, and microbial community structures in drinking water distribution pipes. J. Environ. Manag..

[151] Dalmau-Soler J., Ballesteros-Cano R., Boleda M.R., Paraira M., Ferrer N., Lacorte S. (2021). Microplastics from headwaters to tap water: Occurrence and removal in a drinking water treatment plant in Barcelona Metropolitan area (Catalonia, NE Spain). Environ. Sci. Pollut. Res..

[152] Tong H., Jiang Q., Hu X., Zhong X. (2020). Occurrence and identification of microplastics in tap water from China. Chemosphere.

[153] Cherian A.G., Liu Z., McKie M.J., Almuhtaram H., Andrews R.C. (2023). Microplastic Removal from Drinking Water Using Point-of-Use Devices. Polymers.

[154] Gopakumar A.N., Ccanccapa-Cartagena A., Bell K., Salehi M. (2024). Development of crosslinked polyvinyl alcohol nanofibrous membrane for microplastic removal from water. J. Appl. Polym. Sci..

[155] Li Q., Lai Y., Yu S., Li P., Zhou X., Dong L., Liu X., Yao Z., Liu J. (2021). Sequential Isolation of Microplastics and Nanoplastics in Environmental Waters by Membrane Filtration, Followed by Cloud-Point Extraction. Anal. Chem..

[156] Bai Y., Shan F., Zhu Y.Y., Xu J.Y., Wu Y.S., Luo X.G., Wu Y.U., Hu H.Y., Zhang B.L. (2020). Long-term performance and economic evaluation of full-scale MF and RO process—A case study of the Changi NEWater Project Phase 2 in Singapore. Water Cycle.

[157] Payment P., Franco R., Richardson L., Siemiatyck J. (1991). Gastrointestinal health effects associated with the consumption of drinking water produced by point-of-use domestic reverse-osmosis filtration units. Appl. Environ. Microbiol..

[158] Capodaglio A.G. (2024). Urban Water Supply Sustainability and Resilience under Climate Variability: Innovative Paradigms, Approaches and Technologies. ACS ES&T Water.

